# Use of Targeted Amplicon Sequencing in Peanut to Generate Allele Information on Allotetraploid Sub-Genomes

**DOI:** 10.3390/genes11101220

**Published:** 2020-10-18

**Authors:** Roshan Kulkarni, Ratan Chopra, Jennifer Chagoya, Charles E. Simpson, Michael R. Baring, Andrew Hillhouse, Naveen Puppala, Kelly Chamberlin, Mark D. Burow

**Affiliations:** 1 Department of Plant and Soil Sciences, Texas Tech University, Lubbock, TX 79409, USA; roshank@iastate.edu; 2 Plant Stress and Germplasm Development Unit, United States Department of Agriculture-Agricultural Research Service, Lubbock, TX 79415, USA; rchopra@umn.edu; 3 Texas A&M AgriLife Research and Extension Center, Lubbock, TX 79403, USA; jcchagoya@ag.tamu.edu; 4 Texas A&M AgriLife Research and Extension Center, Stephenville, TX 76401, USA; c-simpson@tamu.edu; 5 Texas A&M AgriLife Research, College Station, TX 77843, USA; m-baring@tamu.edu; 6 Department of Veterinary Medicine and Biomedical Sciences, Texas A & M University, College Station, TX 77843, USA; hillhouse@tamu.edu; 7 Agricultural Sciences Center, New Mexico State University, Clovis, NM 88101, USA; npuppala@nmsu.edu; 8 USDA-ARS, Wheat, Peanut and other Field Crops Research, Stillwater, OK 74075, USA; Kelly.Chamberlin@ars.usda.gov

**Keywords:** targeted resequencing, allopolyploid, heterozygous SNP calls, tetraploids

## Abstract

The use of molecular markers in plant breeding has become a routine practice, but the cost per accession can be a hindrance to the routine use of Quantitative Trait Loci (QTL) identification in breeding programs. In this study, we demonstrate the use of targeted re-sequencing as a proof of concept of a cost-effective approach to retrieve highly informative allele information, as well as develop a bioinformatics strategy to capture the genome-specific information of a polyploid species. SNPs were identified from alignment of raw transcriptome reads (2 × 50 bp) to a synthetic tetraploid genome using BWA followed by a GATK pipeline. Regions containing high polymorphic SNPs in both A genome and B genomes were selected as targets for the resequencing study. Targets were amplified using multiplex PCR followed by sequencing on an Illumina HiSeq. Eighty-one percent of the SNP calls in diploids and 68% of the SNP calls in tetraploids were confirmed. These results were also confirmed by KASP validation. Based on this study, we find that targeted resequencing technologies have potential for obtaining maximum allele information in allopolyploids at reduced cost.

## 1. Introduction

Marker-assisted breeding often requires a large number of samples to be genotyped, especially in early generations, for effective selection [1,2]. Molecular markers such as SSRs and SNPs are the most commonly utilized types for marker-assisted selection. With the advent of NGS (Next Generation Sequencing) technology, high-throughput platforms have been used for SNP identification and genotyping in plants. However, the complexity of the polyploid plant genome has complicated the interpretation of SNP calls, and decreased validation success [3]. Challenges in genotyping polyploids have been reported before [4,5]. Bertioli et al. [6] also reported decreased SNP validation success from genotyping studies in peanut. Moreover, despite a reduction in cost per SNP, the large number of accessions to be genotyped makes the cost of genotyping a population for QTL analysis problematic for many breeding programs.

Cultivated peanut makes an ideal system for testing resequencing in a complex polyploid. *Arachis hypogaea* L., along with the closely-related wild tetraploid species *A. monticola*, is allotetraploid (2n = 4x = 40), wherein two related genomes are present. *A. hypogaea* is widely considered to be derived from a single recent hybridization event between the two diploid species *A. duranensis* and *A. ipaënsis*, followed by chromosome doubling to form a polyploid [7,8,9,10]. However, there is also evidence for limited recombination between genomes near the telomeres, resulting in a limited degree of segmental allopolyploidy [11]. A and B genomes have undergone relatively few changes since polyploidization and thus are very closely related to each other [10,12]. The high similarity between the two genome sequences makes it difficult to differentiate homoeologous from homologous variation. The total genome size of the cultivated peanut is estimated to be around 2.7 G bp with a repetitive content of 64% [10,13,14]. Despite using sophisticated bioinformatics tools, homologous sequences obtained by NGS cannot be separated easily from homoeologous and paralogous sequences, and SNP identification using NGS in allopolyploids has had low validation rates [15]. The software program SWEEP (Sliding Window Extraction of Explicit Polymorphisms) has been developed and implemented to filter out homoeologous SNPs in peanut [16]. SWEEP uses genome polymorphism haplotypes to filter out homoeologous SNPs as a prelude to identifying true homologous SNPs between genotypes.

SNP genotyping methods mentioned above have been useful at identifying or measuring of variation in peanut. The KASP (LGC Ltd., Teddington, UK) assay that is based on single allele discrimination is used for MAS (Marker Assisted Selection) where there are many individuals x a few numbers of SNPs to identify. Genotype by sequencing and SNP chip are cost-effective when there are few individuals ×x thousands of SNPs to screen [17,18]. However, GBS and the SNP chip are expensive for performing QTL analysis for a breeding program. There is a need for the development of a cost-effective method, as low as $5 to $15 per sample, for QTL discovery in populations comprising a few hundred individuals.

Targeted resequencing is a method of sequencing regions of interest from the genome. Sequencing a subset of the genome is performed to lower the costs relative to whole genome sequencing and can be effective of detecting low levels of variation due to higher read depth. Several studies for targeted resequencing have been reported in plants, especially polyploids [19,20,21,22]. In this work, we report the use of the Fluidigm Access Array system for targeted resequencing as a proof of concept in peanut. The Access Array system has been developed by Fluidigm for targeted resequencing using a four-primer system that consists of one pair of nested forward and reverse specific primers, plus a pair of primers containing adapters for Illumina sequencing and barcodes, which are unique tags comprising short sequences and are attached to the end of each DNA sample. These barcodes are used as the identifiers for each DNA sample after pooling for mass sequencing. The Access Array uses an Integrated Fluidic Circuit (IFC) plate, consisting of a silicon chip in the center and made of nano-sized reaction chambers surrounded by 48 wells for primers and 48 wells for DNA samples on the other side. Depending on the chemistry version used, 10–50 plex targets can be used, allowing from 480 to 2400 targets to be run simultaneously. The pooled PCR product is ready for Quality Control (QC) analysis and Illumina sequencing without an additional library preparation step (http://www.fluidigm.com). High read depth of targeted resequencing coupled with multiplexing could potentially be used for cost-effective QTL analysis in peanut and other allopolyploids.

In the current work, we tested targeted resequencing as a potential cost-effective method for QTL analysis in peanut. Forty-eight SNP targets were used, and 48 accessions were evaluated by multiplexed PCR amplification of targets, followed by mass pooled sequencing. We demonstrate that bioinformatic analysis of sequence products was able to validate target SNPs.

## 2. Materials and Methods

### 2.1. Identification of SNPs

**Figure 1:**
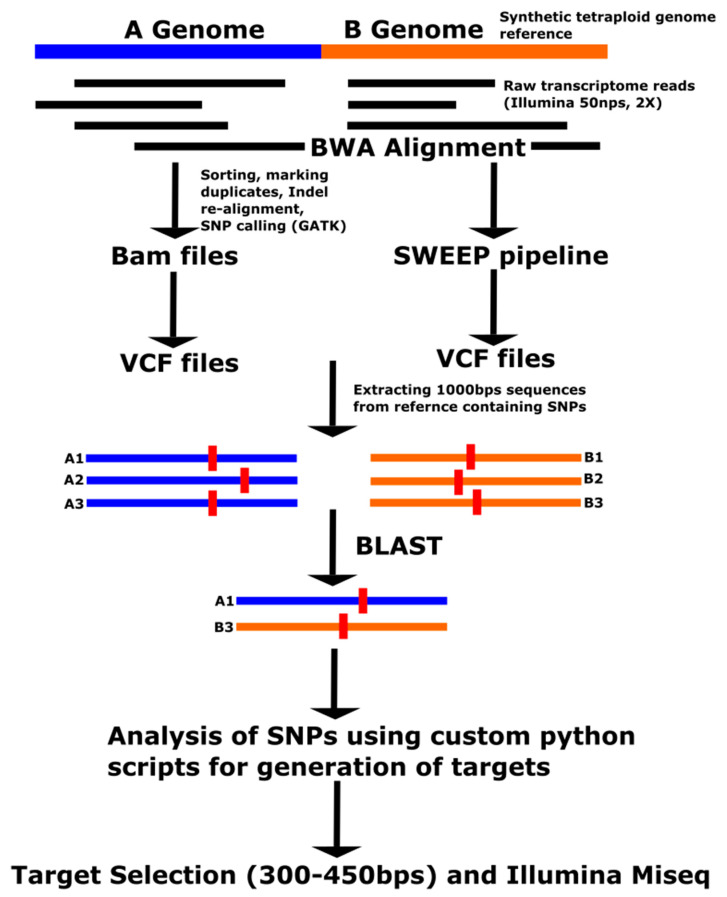
GATK pipeline for SNP identification and target selection (for the OLin method, the SNP identification pipeline would be the same expect for the alignment with the OLin transcriptome sequence instead of the synthetic tetraploid genome reference).

Four approaches were used to select SNP datasets, with different potentials for understanding the peanut tetraploid genome (Table 1). The first set of SNPs was obtained from alignment of raw transcriptome reads (2 × 50 bp) to a synthetic tetraploid genome (Figure 1). This approach is referred to as GATK in the following text. Raw transcriptome reads were used from 10 tetraploid accessions (Supplementary Table S1), which were sequenced previously on an Illumina Ga IIx or HiSeq 2500 by Chopra et al. [23,24]. A synthetic tetraploid genome reference was generated by concatenating *A. duranensis* V14167 and *A. ipaënsis* GKPSSc 30076 diploid genome sequences (pre-release sequences builds) from PeanutBase [10,25]. The alignment was performed using the Burrows-Wheeler Aligner (BWA) version 0.6.2 [26] with standard output as BAM (Binary Alignment Map) files. BAM files were processed using Picard Tools-1.91 (http://broadinstitute.github.io/picard). BAM files were ordered based on the position coordinates using SortSam from Samtools [27]. PCR duplicates were removed using MarkDuplicates, and an index was created using the BuildBamIndex. RealignerTargetCreator and IndelRealigner (Picard Tools- 1.91) were used for realignment of the sequences to correct for gaps due to InDels. SNPs were called using the UnifiedGenotyper program within the Genome Analysis Tool Kit (GATK) 2.7-4 from the Broad Institute [28] with standard output as Variant Call Format (vcf) files. All of the bioinformatics analyses were performed either on Centos or Redhat (Raleigh, NC, USA) Linux operating systems.

The second set of SNPs was obtained using the SWEEP filter version 1 [16] to filter out homoeologous SNPs. This was performed on the SNPs obtained using the GATK approach. The third set of SNPs was obtained from the alignment of raw transcriptome reads (2 × 50 bp) against the OLin reference transcriptome developed by de novo assembly of transcriptome reads of this cultivar [29], and the following pipeline was similar to the GATK approach. The fourth set of SNPs was selected from the Affymetrix Axiom_Array SNP chip (Thermo Fisher Scientific, Walton, MA, USA) [17,18].

### 2.2. Target Selection for the Fluidigm Access Array

The SNPs obtained from the four approaches mentioned above were filtered for Polymorphic Information Content (PIC) greater than 30% and a read depth of greater than 100 when summed across accessions using a custom python script (Supplementary Table S2). InDels were removed manually using a Microsoft (Microsoft Corp., Redmond, WA, USA) Excel filter. Small sequences of approximately 1000 bp upstream and downstream of the filtered SNPs were extracted from the diploid genome reference scaffolds using the ValidationAmplicon tool of GATK 2.7-4 running under Java (Oracle Corp, Redwood City, CA, USA) v7. These sequences are referred to as SNP-containing sequences in the following text. In order to increase the sequence information sufficiently to separate homoeologs, A and B genome regions were simultaneously amplified and targeted. Homoeologous regions containing SNPs were identified from the A and B genome diploid sequence using NCBI-BLAST and custom python scripts. Further SNP analysis was done using custom python scripts to find regions with multiple SNPs in both A and B genome scaffolds (Supplementary Table S2). These regions were used in target selection. A total of 48 targets were selected from the sequences containing SNPs from the four approaches (Supplementary Table S3). Target size ranged from 300 to 450 bp. A consensus sequence was generated for each of the 48 pairs of homoeologous A and B genome SNP-containing sequences to design primers to amplify both genomes simultaneously. Primers were designed for the targets using the Fluidigm Assay design pipeline (Fluidigm, South San Francisco, CA, USA) by Fluidigm Inc. Specificity of the primers was checked by performing the BLAST searches against both the genomes in PeanutBase (https://peanutbase.org). Primer sequences are given in Supplementary Table S4.

### 2.3. DNA Extraction and Fluidigm Access Array Run

DNA was extracted from seed samples of 48 accessions, which included 12 diploids and 36 tetraploids (Supplementary Table S5). Small seed pieces of about 40 mg each were taken from the non-cotyledonary end for DNA extraction. DNA was extracted from seed pieces using the Qiagen DNeasy kit (Qiagen Inc., Germantown, MD, USA). DNA was quantified on 0.8% agarose gels using phage λ DNA concentration standards. DNA yields ranged from 40 to 50 ng/μL, with an elution volume of 100 μL.

The Fluidigm Access Array protocol was used, and samples were preamplified prior to the amplification step (Supplementary Table S6). Amplification was carried out on a Fluidigm Access Array at the Texas Tech University Health Sciences Center (Supplementary Table S6B–F) using the manufacturer’s protocol. The 20× Assay Pools consisted of multiplexed primer sets, containing 2.5 µM of each forward primer and 833 nM each reverse primer. The concentrations of forward and reverse primer in the 20× assays were 1.0 µM and 333 nM, respectively. The final concentrations of forward and reverse primers in the reaction chamber were 50 nM and 16.7 nM, respectively. Final quality control was run on the purified product on a Bioanalyzer 2100 (Agilent, Santa Clara, CA, USA) using the Agilent DNA 1000 kit before sequencing; the minimum product size of the sum of target region and length of barcode primers (300 bps) was considered satisfactory. Samples were pooled and flanking Illumina adapters were added.

### 2.4. Sequencing of Amplicons

Pooled amplicons from the Fluidigm Access Array were sequenced on an Illumina MiSeq at the College of Veterinary Medicine, Texas A&M University, College Station, TX using paired-end (2 × 250 bp) sequencing. PhiX174 DNA was added to 5% final concentration relative to the other DNA samples to make up for low diversity libraries. Samples were demultiplexed by identification of barcodes.

### 2.5. Analysis and Validation

Bioinformatic analyses were performed using the Texas Tech University Centos based Hrothgar High Performance Computing Cloud (HPCC) or on laboratory servers running Red Hat Enterprise Linux or CentOS 6.7. Sequencing results from the MiSeq consisted of 48 files, which were produced by demultiplexing by barcode sequence, each plant having a unique barcode. Each file included sequences from the 48 targets for that plant sample. Forward and reverse reads from each file were combined using FLASH [30]. The file of merged reads was aligned to the synthetic tetraploid genome reference sequence. In addition, the 48 A and B genome target sequences were pooled for use as a second reference set of target sequences for alignment of reads from the MiSeq; this was done to avoid paralogous matches and possible false SNP calls. Merged forward and reverse MiSeq reads were then mapped against the newly generated subset reference of 48 target sequences to identify SNPs. Alignments were performed using the BWA aligner, and the SNPs were called using the GATK pipeline mentioned earlier with coverage depth increased to 4000 for maximum representation of reads, as the default coverage depth is 250 for GATK. SNPs were called separately against the whole genome reference and the pooled 48 target reference sequences. SNPs calls were subjected to 15, 20, and 25% minimum allele frequency thresholds for the minor allele to reduce the rate of calling of false SNPs. SNPs called from targeted resequencing were also compared against the original SNP calls from transcriptome data for validation.

### 2.6. KASP Validation

A subset of eight targets representing all four approaches was selected from the original 48 targets for validation using Kompetitive Allele-Specific PCR (KASP) assays. For this, a subset of 24 genotypes representing all four market types plus selected wild species was selected from the original set of 48 genotypes. The KASP assay was performed on a Lightcycler 480 (Roche Inc., Indianapolis, IN, USA) using filters for dyes FAM and HEX dyes in the master mix. The PCR conditions and primer sequences are given in Supplementary Tables S7 and S8.

### 2.7. Sequencing Cost

Sequencing cost was estimated in three ways, two using the Fluidigm Access Array. The first used the Fluidigm Access Array instructions (Fluidigm, 2012) and prices of 2012; in this, primers were designed and ordered from IDT (Coralville, IA, USA), and reagents except the barcode set were ordered from third parties. The chemistry was limited in ability to multiplex samples up to 10-fold, as per the manufacturer’s instructions. In the second, the updated Fluidigm kit was used, with newer chemistry and all reagents purchased from Fluidigm; likewise, primers were designed and synthesized from Fluidigm (Fluidigm, South San Francisco, CA, USA); current prices were used. Multiplexing was allowed up to 50-plex. Results from this method are presented below. In the third, samples were all custom designed as per Shirasawa et al. [22] and amplifications up to 50-plex in 384 well plates. All reagent prices were calculated using third party suppliers. Costs were estimated as reagents plus design services; labor cost was not estimated.

## 3. Results

### 3.1. SNP Identification

From the GATK pipeline, approximately 1.6 million raw SNPs were identified from the SNP identification pipeline (Table 2). These SNPs were filtered for a minor allele frequency of more than 10%, a read depth of greater than 100 summed across 10 accessions, and Polymorphic Information Content (PIC) of greater than or equal to 0.3. After the filter, 9884 SNPs from 617 A-genome scaffolds and 5684 SNPs from 184 B-genome scaffolds were obtained from tetraploids. Among these, 10,533 SNPs from 418 scaffolds were found to be in the corresponding regions of A genome diploids and also the A genome of the tetraploids, and 502 SNPs were found from 129 genome scaffolds in the homoeologous regions of A genome diploids and A and B genome tetraploids. In the case of the A genome, a greater number of SNPs were present in the diploid/tetraploid comparison than in the A genome of the tetraploids because of the additional SNPs among A genome diploids on the same region that included 250 bp of flanking sequence in each direction surrounding the A-genome tetraploid SNPs. Raw SNPs were also filtered through the SWEEP program. About 40% to 50% of the SNPs identified from GATK were retained by the SWEEP program. SNPs present in the similar regions of A genome diploids and both genomes of tetraploids were used for further target selection. From the alignment with the OLin tetraploid transcriptome reference, 865 SNPs were identified with polymorphic information content greater than 0.3 and with read depth of greater than 100 (Table 3).

### 3.2. MiSeq Sequencing

**Figure 2:**
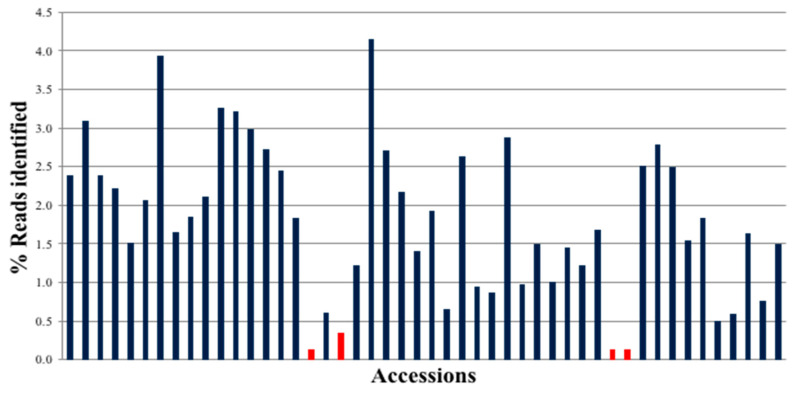
MiSeq Sequencing Run Distribution of reads by Peanut Accession on MiSeq (250X2) (total reads: 30,153,796; PF Reads (Reads that have passed Illumina quality filter): 24,430,536) (each index corresponds to a different plant accession, given in Supplemental Table S5). Red bars indicate the accessions that failed to amplify satisfactorily.

The Illumina MiSeq run for 48 samples × 48 targets generated 30,153,796 reads, of which 24,430,536 were identified with barcodes (Figure 2). Amplified sequences from all peanut accessions were present, and most of them had similar representation of reads. The exceptions were three samples that had low read depth due to the introduction of bubbles when loading onto the 48.48 IFC.

### 3.3. SNPs Analysis and Validation

Among the four methods of SNP identification and selection compared, the three methods based on use of a genomic reference gave the highest percentage of validation. GATK had 52.6% of the total polymorphic targets, and 71.4% of SNPs in targets that were successfully identified by sequence analysis validated in the MiSeq run; targets obtained from the SNP chip had 66.7% polymorphism and 66.7% validation, and SNPs filtered through SWEEP had a polymorphism rate of 55.5% and a validation ratio of 62.5%. Targets based on the OLin transcriptome reference [29] had the lowest polymorphism and validation rates (28.5% and 50%, respectively) (Table 4).

In the first approach for compiling reads, reads were mapped to the pooled target reference of 48 target regions, and 111 SNPs were identified among the 35 tetraploid accessions. These mapped reads represented 25 genome scaffolds (target regions), as there were many cases where two targets were on a single scaffold. Among these 111 SNPs, 81 SNPs were highly polymorphic with PIC values higher than 0.3 (Table 5). In contrast to the tetraploids, 246 SNPs representing 38 scaffolds were polymorphic among the four A genome diploid accessions (*A. duranensis* KSSc38901, *A. duranensis* K7988, *A. cardenasii* GKP10017 and *A. diogoi* GK10602). Among the four B genome diploid accessions (*A. ipaënsis* GKPSSc30076, *A. magna* GKSSc30097, *A. cruziana* KSSc36024 and *A. batizicoi* K9484), 313 SNPs representing 39 scaffolds were highly polymorphic.

In the second approach, reads were mapped to the whole genome reference, from which 73 SNPs were identified among the 35 tetraploid accessions, representing 22 contigs. Among these 73 SNPs, 56 SNPs were highly polymorphic with PIC values higher than 0.3. Among the four A genome diploid accessions (*A. duranensis* KSSc38901, *A. duranensis* K7988, *A. cardenasii* GKP10017 and *A. diogoi* GK10602), 207 SNPs were highly polymorphic. Among the four B genome diploid accessions (*A. ipaënsis* GKPSSc30076, *A. magna* GKSSc30097, *A. cruziana* KSSc36024 and *A. batizicoi* K9484), 262 SNPs were highly polymorphic.

In general, a large number of SNPs identified from all the four approaches of initial SNP identification were called as heterozygous (Supplementary Table S9). Alignment of MiSeq sequences to the synthetic tetraploid reference resulted in a mean of 11.1% heterozygous SNP calls for diploids and 7.5% for tetraploid accessions. Histograms showing SNP distribution were plotted for each accession. Histograms for some diploid and tetraploid accession are shown in Supplementary Figure S1. Based on these results, it was estimated that a range between 30% to 70% reference allele accurately denoted true heterozygotes in diploids. Peanut is a self-pollinating species, thus, for diploids we do not expect many heterozygotes. Few heterozygotes are expected for tetraploids; however, sequences of homoeologs could have co-mapped and would be considered heterozygotes. Lower percentage heterozygote values were suspected to be incorrect, and the percentage of heterozygotes called based on unfiltered calls varied substantially by accession—from a low of 3.3% in the B genome diploid *A. ipaënsis* GKPSSc30076 to 21.2% in the K genome diploid *A. batizicoi* K9484. Among tetraploids, the range of heterozygotes called varied from 4.6% (CC 155A) to 24.4% (TxAG-6). After correction for a 20% heterozygote threshold, the percentage of heterozygotes ranged from 0.1% to 0.3% (Supplementary Table S9).

### 3.4. KASP Validation

**Figure 3:**
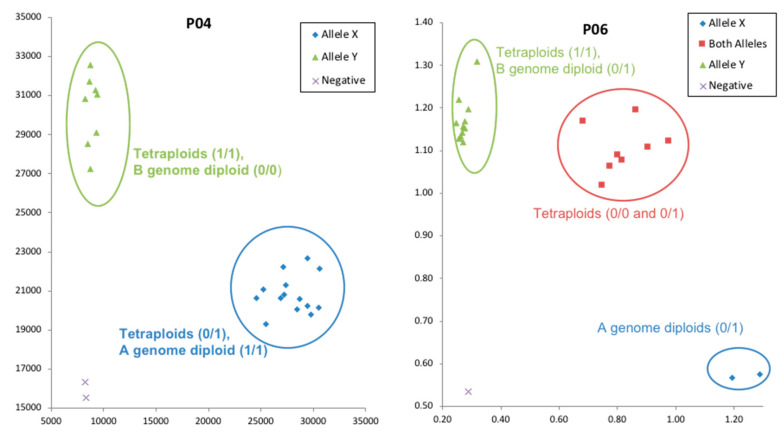
Example of validation of SNP calls derived from MiSeq using KASP markers.

SNP calls obtained from the KASP validation experiment for eight selected targets for 24 genotypes were compared with SNP calls from resequencing for further validation. One targeted was monomorphic according to the KASP assay, while for the other seven targets, 86% of the SNP clusters from the KASP assay matched the SNP calls obtained from resequencing (Table 6). An example of KASP validation for two primers is shown in Figure 3.

**Figure 4:**
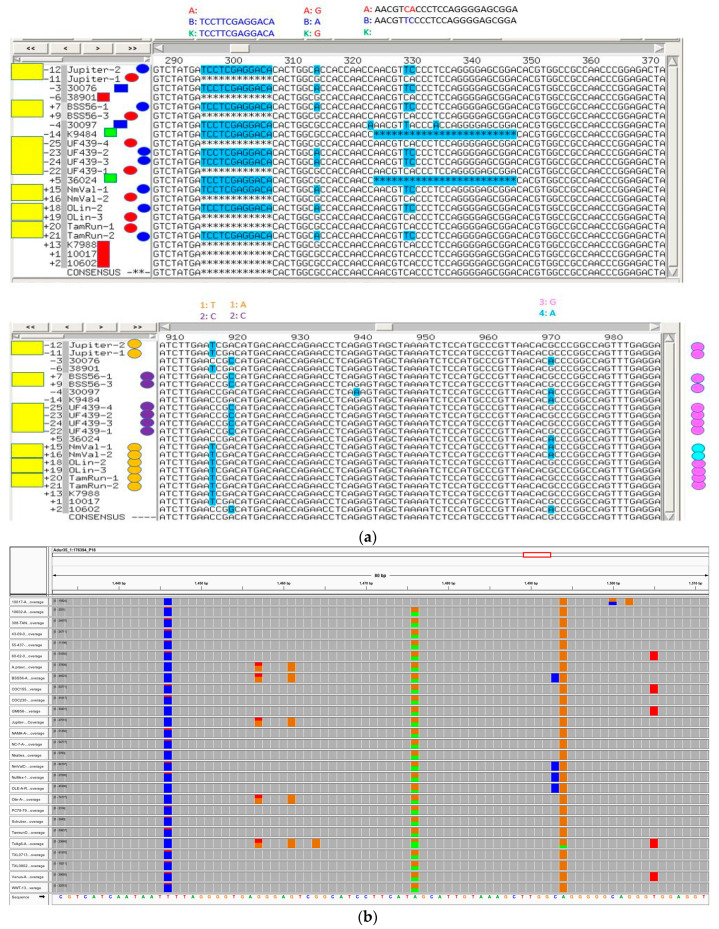
Target sequence with multiple SNPs and indels. (**a**)—An example showing homoelogous (top) and homoeologous (bottom) SNPs in tetraploids. Yellow rectangles at left denote tetraploids. Red, blue, and green rectangles (top) denote A, B, and K genome diploids, and red and blue ovals denote A and B genomes in tetraploids. Allele calls are shown above the figure. At bottom are homologous SNPs differentiating tetraploids; SNPs are denoted above the figure as alleles 1 or 2 (orange and purple ovals at left), and 3 or 4 (pink or cyan ovals at right). The order of sequences is the same in the top and bottom figures. (**b**) An example of target sequence in IGV viewer showing multiple SNPs (There are two SNPs showing up in all accessions (blue and orange), which may be referred to as anchor SNPs). The target SNP is highlighted by a red circle.

A principal components analysis plot using TASSEL 5.0 [31] demonstrated a clear separation of clusters between A-genome and B-genome diploid accessions. Tetraploid accessions clustered out separately based on the botanical types hypogaea and fastigiata as expected (Supplementary Figure S2). Homoeologous SNPs in tetraploids could be differentiated by aligning tetraploid sequences with A genome and B genome diploid sequences and viewing in Integrative Genome Browser (IGV) (Figure 4).

### 3.5. Sequencing Cost

Depending on the time and method of analysis, prices were at times competitive vs. RADSeq or the peanut Axiom_Array SNP chip [18]. From 2016 to 2018, prices for RAD-Seq were $50/sample at the Texas A&M AgriLife Bioinformatics and Genomics Center, and the same price was indicated for the Axiom_Array SNP chip. Using the 2012 version of the Fluidigm manual and third-party prices, the per plant accession cost of running a 48-sample chip was $28.75 (Table 7). The cost was substantially higher, $77.60, if all reagents and primers were purchased from Fluidigm using the 2016 kit; the greatest difference was in the cost of the primers −45% of the total cost was for purchase of the primers. By contrast, cost of running the samples on 384-well plates using all generic components was estimated as $17.72 per sample.

For an experiment involving 384 plant samples and 960 amplicon targets, costs were approximately 20% lower per sample, with prices as low as $23.64/sample (2012 kit) for the Access Array and $14.08 using all generic components (Table 8).

Comparing to other means of analysis, prices per sample for resequencing ($28.75) were lower using the 2012 instructions than the prices for RADSeq and the SNP chip in the 2016–2018 time frame. Since then, the prices for the latter two have dropped to $28/sample for RADSeq and the Axiom_Array2 chip. The Fluidigm prices chemistry changed in 2016 to allow higher multiplexing, but the newer protocol dropped instructions on primer design from third parties; the revised prices at $66/sample are higher than other means of analysis. However, we have estimated that running the analysis using generic components in 384-well plates can be as low as $14/sample, half the cost of other analyses, but less convenient than using the Access Array.

The current experiment was designed as a proof of concept, to test resequencing in peanut, and also to determine whether it could provide a lower cost method of genotyping, at a marker density lower than high throughput methods, but sufficient for QTL analysis and marker-assisted selection. Ultimately, a price point of $5/sample would be ideal for breeding programs. The current work suggests that we do not have the ability to perform this at that price; however, it may be possible to drop the price by 50% compared to other methods. This would still be useful. The major costs of such an approach are primer synthesis and sequencing. Lower primer costs are possible; they have been estimated at the 500pmol scale and $0.10/base. Smaller-scale syntheses could reduce costs by 75% at 10 pmol to 50 pmol scales, but the volumes needed for PCR reactions in 384-well plates would not make it possible to synthesize sufficient primer amounts without miniaturization to use the IFCs; however, the costs of the IFCs is greater than the cost savings that could be realized. Throughput of newer sequencers is higher, but the higher cost per run offsets much of the advantage.

## 4. Discussion

The re-sequencing effort with three approaches, GATK, SWEEP and SNP chip, was successful, with 81% of the targets for the diploids and 68% of the targets for tetraploids being found polymorphic by resequencing (Table 4). However, a much lower percentage of targets selected using the OLin tetraploid transcriptome reference could be validated. Although the sequences were aligned against the older genome sequence assembly (March 2014), they were validated based on the newer version of the genome assembly (February 2016). The difference in the versions of the reference sequences might be the reason for the inconsistent mapping of the OLin based targets, or lower completeness of the transcriptome identification assembly may be responsible. This suggests that the use of a genome sequence reference is essential for accurate SNP target selection. KASP assays were designed for some of the targets from targeted resequencing as a validation of results. In general, most but not all of the SNP call patterns from KASP assays agreed with the SNP calls obtained from MiSeq.

Three potential difficulties could explain the low (68%) agreement between resequencing and the initial transcriptome sequence results. The first is that SNPs from the GATK, SWEEP and OLin methods were developed from a transcriptome sequence. Some of the SNP targets may have been spurious, a result of lack of expression of some genes; when resequencing or KASP assays were performed, the genotyping was based on genomic DNA and not RNA expression. However, SNP targets selected from the SNP chip, identified by whole genome sequencing, were not more accurate. A second possible explanation is polyploidy; indeed, difficulty in tetraploid SNP validation has been reported before [6]. Lack of success in validating tetraploid SNPs could possibly be due to difficulty in resolving A and B homoeologs. For most of the heterozygous SNP calls, 0/1 scores were obtained, but it was not always possible to assign the 0/0 or 1/1 score to a particular genome. This issue might be due to several reasons: firstly, BWA aligner is not designed to handle tetraploid alignments, and the targets chosen for SNP validation from both the sub-genomes differed only by 4–5 nucleotides [32,33,34]. Homoeolog sequence differentiation is difficult due to such minimal differences. Thirdly, sequence homology was assumed from BLAST results based on diploid genome sequences. The presence of paralogs and other copies that may be present in the diploid and tetraploid genomes may shift the mapping of sequences from expected targets [13].

### 4.1. Source of Superfluous Heterozygote Calls

Variant call files (vcf) of sequences from analysis of MiSeq data had heterozygous SNP calls with means of 11.1% for diploids and 7.5% for tetraploids. These were too low to be likely for any given locus, as 50%, called heterozygosity, is expected for a true diploid heterozygote, or for a pair of tetraploid homoeologs wherein the allele calls for A and B genomes were different but each was homozygous. The observed mean values are too high for the set of targets overall as the peanut accessions are considered to be self-pollinated lines. Diploid accessions not only lack homoeologs, but many are incompatible with each other, and thus, could not be the products of recent hybridization.

Three reasons can be hypothesized for the apparent unexpectedly high proportions of heterozygous allele calls: (1) sequencing error, (2) inaccurate mapping to the A- or B-genome in tetraploids, resulting in overlap of homoeologs or (3) co-mapping of paralogs. Each of these possibilities was considered.

(1) Sequence data used a minimum quality (Q) score of 30, thus, an error rate of less than 0.1% might be expected, far less than the heterozygote percentage in the range of 7 to 11% that was observed. A study on MiSeq error profiling suggests that errors from MiSeq sequencing are not random and are more prone to G/A and A/C base substitutions, and there is an increase in sequencing error towards the end of the sequence [35]. To estimate the error percentages from MiSeq data, error percentages for each base pair substitution from barcode regions of accessions OLin and Jupiter were calculated (Supplementary Table S10). G/A substitution had the highest error rate (1.38%) followed by A/T substitution with an error rate of 0.84%. These estimates were substantially lower than the observed rate of heterozygous calls, and therefore, were not the major factor contributing to rate of heterozygous calls. Nevertheless, it was evidence that the reported quality values were overestimated.
(2) Even though we expect all A genome reads to map against the A genome part of the synthetic tetraploid reference, and all B genome reads to map against B genome part, there is little control over the alignment due to the fact of high similarity between the two genomes. Additionally, the BWA aligner used in this analysis is not a specialized tetraploid aligner and the power for BWA aligner to discern the few differences between the sub-genomes is unclear. However, even though homoeologous sequences were not completely resolved, they were distinguished to some extent by aligning these sequences against corresponding A and B genome diploid sequences (Figure 4A). Arguing against incomplete resolution of homoelogs was that a high frequency of heterozygous SNP calls was observed among diploid accessions, where two genomes are not present. In the .vcf files, 3.3%, 9.0% and 21.2% heterozygous SNP calls were observed for *A. ipaënsis* GKPSSc30076, *A. duranensis* GKP10017 and *A. batizicoi* K9484, respectively.
(3) Due to the highly-repetitive nature of the peanut genome, reads co-mapping to paralogous sequences could contribute towards apparent heterozygosity. To check the presence of paralogs, target primer sequences were compared against the A diploid genome using BLAST (Supplementary Table S11). There were 183 putative paralogous matches present for 48 targets with an average of 3.81 possible paralogous matches per target. An example of a CLUSTAL alignment of a paralogous match for one of the targets is shown in Supplementary Table S12.

If paralogs were a major contributing factor to apparent heterozygosity, the rate of heterozygous SNPs in the tetraploids would be expected to be similar to the average of the two diploid parents. An expected frequency of SNP heterozygous calls for UF439-16-10-3-2 could be estimated as 6.2%, and was observed to be 5.7%. Similarly, estimation of heterozygous calls of TxAG-6 (*A. cardenasii* GKP10017, *A. diogoi* GK10602 and *A. batizicoi* K9484) was 16.0%, while the observed rate for TxAG-6 was 24.4%.

### 4.2. Interpretation and Correction of Calls

Based on observation of the histograms (Supplementary Figure S1), it was estimated that a range between 30% to 70% reference allele denoted true heterozygotes in diploids, or homozygous homoeologs for tetraploids. This was based on the presence in *A. duranensis*, *A. diogoi* and *A. batizicoi* of peaks in the range of circa 40% to 65% heterozygosity. In addition, there were also peaks between 20% and 30% heterozygosity (*A. batizocoi*) or between 70% and 80% heterozygosity (*A. duranensis*, *A. diogoi*, and *A. batizicoi*). Peaks in these three ranges also occurred in the tetraploids TxAG-6 and UF439-16-10-3-2. These were explained as follows: In the case of diploids, a peak of 50% would indicate a true SNP in one gene. The expected percentage heterozygosity would be 50%. In cases of tetraploids, this range can be considered to correspond to cases of a homozygous SNP distinguishing A- and B-genome homoeologs of that accession. In the case of a heterozygous SNP in one genome while the homoeologous allele was homozygous, 25% or 75% would be called heterozygous. In the case of two paralogous genes present, an SNP in one would result in a peak at 25% or 75% assuming equal representations of both genome sequences. This would correspond to the peaks observed in the 20% to 30% range and in the 70% to 80% range for the tetraploids. For diploids, this could represent presence of two paralogs, one homozygous and the other heterozygous and sharing one target nucleotide identical with the homozygous paralog.

The original SNP call dataset was divided into three sub-datasets using cutoff values of 25%, 20% and 15% correction for the minor allele using python scripts. If the percentage of heterozygotes in the minor allele was less than the cutoff value, the minor allele was rescored as a homozygote instead of the heterozygote. We observed validation values of 85.4%, 85.7% and 80.5%, respectively, for 25%, 20% and 15% cutoff-corrected SNP calls. With less correction (15% cutoff), there was a lower validation rate, but the 20% cutoff would allow for identification of true heterozygotes in tetraploids while not reducing the validation rate. This correction reduced an average of heterozygous SNP calls to 0.18% from the initial 8.5% (Supplementary Table S1). Minor allele frequencies, less than 20%, were deemed to result from a combination of the presence of either two paralogous sequences containing the target region sequence but one present at a higher frequency, or possibly three or more paralogous sequences. Additional contributing factors could include the higher-than-expected sequencing error rate, and/or misidentification of homoeologous sequences. There are several instances where the distortions in the SNP calls are reported in tetraploids and previous reports using other marker types have likewise reported segregation distortion in tetraploid peanut [6,36]. Peace et al. [37] reported the presence of heterozygous SNPs and the inability to resolve the dosage between the sub-genomes in sweet cherries.

In addition to scoring targeted SNPs, sequencing also identified some non-targeted SNPs present in the surrounding regions. In the case of accessions of *A. hypogaea*, these were approximately double the number of the targeted SNPs validated; for the diploids, the numbers were much higher. Such SNPs may be in linkage disequilibrium [38] and may not provide any novel variant information. However, these SNPs can be used to identify conserved haplotypes or to distinguish A and B genomes.

Given that paralogs were found for 37 of 48 targets (Supplementary Table S11), it is probably not possible to devise a complete set of highly-polymorphic targets without corresponding paralogs. However, selecting targets with relatively-few paralogs may be worthwhile. Additionally, more-accurate analysis of targeted resequencing may be achieved using the newly released tetraploid assembly [14]. Moreover, a newer statistical approach involving likelihood-based methods is under development (Kulkarni et al., in preparation) [39], and is expected to improve scoring accuracy. Together, an accurate automated pipeline for SNP calling can be developed.

## 5. Conclusions

The current experiment was designed as a proof of concept, using the Fluidigm Access Array for multiplexing, to demonstrate the possibility of resequencing in peanut, and also to determine whether it could provide a lower cost method of genotyping, at a marker density sufficient for initial QTL analysis and marker-assisted selection. Validation of results obtained from targeted resequencing suggests that targeted resequencing could be used for QTL mapping studies. For QTL identification, a set of 500 to 2000 highly-polymorphic SNPs can be selected that could potentially be used for any cultivated x cultivated cross. Amplification of targets from both sub-genomes simultaneously can effectively double the number of target loci, increasing the genomic information that can be extracted. Although a price point of $5–10/sample would be ideal for breeding programs, the current work suggests that it may be possible to drop the price by 50% compared to other methods (GBS, SNP chip). In addition, as much of the price of resequencing is the primer cost, once this is paid upfront, genotyping cost per sample drops by 50% to $7/sample thereafter. We believe such an approach can be readily adopted in many allopolyploid species. GBS or the SNP chip could be used subsequently for high-resolution mapping of the target region for genetic studies, if desired.

## Figures and Tables

**Table 1:** Summary of target selection from different approaches for the Fluidigm experiment (targets selected from GATK, filtered through SWEEP, OLin as reference and SNP chip as control).

Approach	No. of Targets
GATK (Genome reference)	18
SWEEP (Genome reference)	10
OLin (Olin reference)	14
SNP chip (control)	6

**Table 2:** Summary of SNPs identified from the SNP identification pipeline for target design.

Criterion	SNP Comparison	Number of Genome Sequence Scaffolds	Number of SNPs	SWEEP (No. of Genome Sequence Scaffolds)	SWEEP (No. of SNPs)	SNPs in Common to GATK and SWEEP
Raw SNPs	NA	NA	1.6 million	NA	NA	NA
Filter for MAF ^1^	A Genome SNPs among tetraploids	1157	222,387	881	55,234	NA
Filter for MAF	B Genome SNPs among tetraploids	247	64,090	231	16,343	NA
Filter for RD ^2^ (100), PIC ^3^ (0.3)	A Genome SNPs among tetraploids	617	9884	452	6292	4618
Filter for RD ^2^ (100), PIC ^3^ (0.3)	B Genome SNPs among tetraploids	184	5684	152	2548	2128
SNPs from common contigs (+/− 100 bp)	SNPs common to A genome of tetraploids and A genome diploids	418	10,533	309	7153	3004
SNPs from common contigs (+/− 100 bp)	SNPs common to tetraploid A and B genomes and also to the parents of the A genome diploid population	129	502	60	192	179
Best BLAST Hit * (Alignment length > 350 bps)	SNPs common to both A and B tetraploid genomes	NA	517	NA	212	NA

^1^ MAF—Minor Allele Frequency filter > 10%; ^2^ RD—Read Depth; ^3^ PIC—Polymorphic Information Content. ‘*’—BLAST was performed to identify homoeologous SNP containing scaffolds in order to target SNPs common to both A and B genome tetraploids. ‘NA’—Represents either not applicable or it was not calculated for that particular comparison.

**Table 3:** Summary of SNPs identified from the OLin tetraploid reference method.

Read Depth	Polymorphic Information Content (PIC)	Number of SNPs
>100	>0.5	15
>100	0.4–0.49	592
>100	0.3–0.39	258

**Table 4:** Summary of percentage validation of SNPs from different methods.

	Criterion	Method					
		GATK	SWEEP	SNP Chip	OLin	Sum	GATK + SWEEP + SNP Chip
A genome diploid	Total no. of targets	19	.	6	.	25	25
	No. of missing targets	4	.	0	.	4	4
	No. of targets assayed	15	.	6	.	21	21
	No. of targets validated	12	.	5	.	17	17
	% Validation (of assayed)	80.0		83.3	.	80.9	80.9
	% Validation (of targets)	63.1	.	83.3	.	68.0	68.0
Tetra-ploids	Total no. targets	19	9	6	14	48	34
	No. of missing targets	5	1	0	6	12	6
	No. of targets assayed	14	8	6	8	36	28
	No. of targets validated	10	5	4	4	23	19
	% Validation (% of assayed)	71.4	62.5	66.6	50	50	68
	% validation (% of targets)	52.6	55.5	66.7	28.5	47.9	55.8

‘.’—Not targeted.

**Table 5:** Summary of SNPs obtained from MiSeq reads mapped to the 48 targets.

48 Target Region	
Genome	No. of SNPs
Tetraploid	111
Tetraploid (PIC > 0.3)	81
A genome diploid	246
B genome diploid	313

**Table 6:** KASP validation.

Primer No.	Source Details	Validation Percentage
P01	MiSeq sequence derived from GATK compared against 48 target references	73
P03	MiSeq sequence derived from the SNP chip	monomorphic
P04	MiSeq sequence derived from SWEEP	100
P05	MiSeq sequence derived from SWEEP	100
P06	MiSeq sequence derived from GATK compared against 48 target references	73
P07	MiSeq sequence derived from GATK aligned against the whole genome reference	100
P08	MiSeq sequence derived from GATK aligned against the whole genome reference	92
P10	MiSeq sequence derived from GATK aligned against the whole genome reference	70

**Table 7:** Cost analysis for amplicon sequencing for different chemistries for 48 plate reactions.

Item	Fluidigm 2012	Fluidigm 2020	Generic
48.48 IFC	238	477	0
PCR plates	10	10	10
Integrated Reagents Pkg	0	481	0
Loading and other solutions	15	0	0
Primers (48)	730	2400	384
Barcodes	21	0	58
Taq	4	0	6
Beads	5	0	36
Sequencing	357	357	357
-------------	-------------	-------------	-------------
MiSeq 48 samples	1380	3725	850
MiSeq per sample	28.75	77.60	17.72

**Table 8:** Cost analysis for amplicon sequencing for different chemistries for 384 plate reactions.

Item	Fluidigm 2012	Fluidigm 2020	Generic
48.48 IFC	3808	3816	0
PCR plates	80	80	100
Integrated Reagents Pkg	0	3848	0
Loading and other solutions	15	0	0
Primers (960)	2359	14,746	2359
Barcodes	341	0	418
Taq	35	0	971
Beads	73	0	179
Sequencing	2365	1381	1381
-------------	-------------	-------------	-------------
HiSeq/NovaSeq 384 samples	$9076	$23,871	$5408
HiSeq/NovaSeq per sample	$23.64	$62.16	$14.08

## Data Availability

Summary of the datasets generated and/or analyzed during this study are available in supplementary files. The authors confirm that all data underlying the findings are fully available without restriction. Assemblies and variant information described in this manuscript have been deposited at Figshare at the following URL: https://dx.doi.org/10.6084/m9.figshare.c.4846398. SAM files of raw read data for each of the accessions have been deposited in the NCBI SRA database under bioproject PRJNA605472.

## Supplementary Materials

The following are available online at https://www.mdpi.com/2073-4425/11/10/1220/s1: Table S1, List of accessions for which raw transcriptome reads were aligned against the synthetic tetraploid genome; Table S2, Python scripts used for analysis of VCF files; Table S3, Complete list of the targets and accessions used for targeted re-sequencing; Table S4, Complete list of primers used for Access Array experiment; Table S5, Complete list of accessions used for targeted re-sequencing; Table S6, Fluidigm protocols used; Table S7, KASP experiment details; Table S8, KASP primer sequences; Table S9, Summary of percentage heterozygotes in SNP calls; Table S10, Estimated error percentages for MiSeq for each base pair substitution from barcode regions of accessions OLin and Jupiter; Table S11, Blast results for all targets showing mapping against possible paralogous sequence matches; Table S12, Example of Clustal alignment. Figure S1, Histograms showing the distribution of reported heterozygotes; Figure S2, PCA plot clustering tetraploids based on botanical type.
