# Supplementary material for: Use of Targeted Amplicon Sequencing in Peanut to Generate Allele Information on Allotetraploid Sub-Genomes
Source: Genes (Basel). 2020 Oct 18;11(10):1220. doi: 10.3390/genes11101220 (PMC7650781; doi:10.3390/genes11101220)
Supplement: Supplementary file 1 [file genes-11-01220-s001.zip › genes-942386-supplementary/Supplementary_figures.docx]

**Supplementary Figure Legends**.

**Figure S1.** Histograms showing the distribution of reported heterozygotes. Based on these results, it was estimated that a range between 25% to 75% reference allele denoted true heterozygotes. Minor allele frequencies less than 20% were deemed to result from a combination of presence of paralogous sequences containing the target region sequence (Bar represents the region of putative heterozygous SNPs).


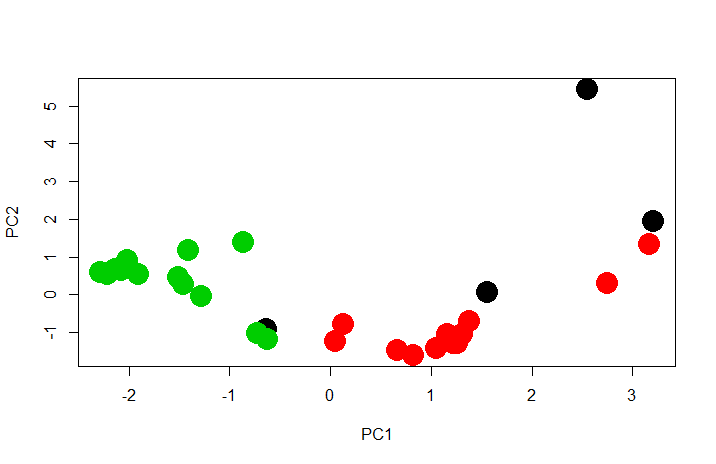


**Figure S2.** PCA plot clustering tetraploids based on botanical type (Green- *hypogaea*, Red- *fastigiata*, Black- Others (TxAG-6, 308-TAN, 60-02-03-03, 43-09-03-02). Note: 60-02-03-03 and 43-09-03-02 are BC_3_ tetraploids from a TxAG-6 x UF439-16-10-3-2 cross.
