# Supplementary material for: Use of Targeted Amplicon Sequencing in Peanut to Generate Allele Information on Allotetraploid Sub-Genomes
Source: Genes (Basel). 2020 Oct 18;11(10):1220. doi: 10.3390/genes11101220 (PMC7650781; doi:10.3390/genes11101220)

**Table S1.** List of accessions for which raw transcriptome reads were aligned against the synthetic tetraploid genome.

| **Accession Number** | **Genotype** | **Reference** |
| --- | --- | --- |
| 1 | BSS56 | [40] |
| 2 | COC155A (PI 502111) | [41] |
| 3 | COC367 (PI 268868) | [41] |
| 4 | COC559 (PI 158854) | [41] |
| 5 | PI648241 | - |
| 6 | PI648242 | - |
| 7 | Jupiter | - |
| 8 | New Mexico Valencia C | [42] |
| 9 | OLin | [43] |
| 10 | Tamrun OL07 | [44] |
|  |  |  |

**Table S2.** Python scripts used for analysis of VCF files

**Script 1: Script for clean-up of Indels (Removing indels from sequence database)**

# This code is to filter out indels

# Importing csv function

import csv # VCF file

#Importing numpy function

import numpy as np

# Opening the destination file

fd = open ('Tetraploid_AGenome_RMindels.txt', 'w')

count = 0

# Opening the target file

with open ('Tetraploid_genome_Agenome_filtered_pic-RD.txt', 'rb') as csvfile:

data = csv.reader(csvfile,delimiter='\t')

for row in data:

s1 = row[3]

s2 = row[4]

# Replacing comma by none for selected rows

ss1 = s1.replace(',','')

ss2 = s2.replace(',','')

# Filtering out rows that have less than 3 characters

if len(ss1) > 3 or len(ss2) > 3:

for i in xrange(0,21):

fd.write((row[i]) + '\t')

fd.write('\n')

fd.close()

**Script 2: Script for calculating PIC**

# This code is for calculating Polymorphic information content (PIC)

# Importing CSV function

import csv

# Importing numpy function

import numpy as np

# Opening the destination file

fd = open ('Tetraploid_BGenome_RMindels_PIC.txt', 'w')

# Opening the target file

with open ('Tetraploid_BGenome_RMindels.txt', 'rb') as csvfile:

data = csv.reader(csvfile,delimiter='\t')

for row in data:

# Assigning count equal to zero for the selected rows

count = []

for i in xrange(13):

count.append(0.0)

# Selection of rows for counting

# Considering all possiblities for counting: '0/0','0/1','1/1','0/2','1/2','2/2','0/3','1/3','2/3','3/3'

for idx in xrange(5,19):

if idx > 5:

sample = row[idx]

fields = sample.split(':')

if fields[0] == '0/0':

count[0] += 1

elif fields[0] == '0/1':

count[1] += 1

elif fields[0] == '1/1':

count[2] += 1

elif fields[0] == '0/2':

count[3] += 1

elif fields[0] == '1/2':

count[4] += 1

elif fields[0] == '2/2':

count[5] += 1

elif fields[0] == '0/3':

count[6] += 1

elif fields[0] == '1/3':

count[7] += 1

elif fields[0] == '2/3':

count[8] += 1

elif fields[0] == '3/3':

count[9] += 1

else:

fd.write(row[idx] + '\t')

# Using the following formula to calculate PIC

count_N = np.divide(count, 13)

pic = 1 - np.sum(np.square(count_N))

fd.write(str(pic) + '\t')

fd.write('\n')

fd.close()

**Script 3: Script to identify SNPs common to A genome diploids and A genome tetraploids (+/- 100 bps)**

# This code is to filter out SNPs common to A genome diploids and A genome tetraploids (+/- 100 bps)

import csv

import numpy as np

fd = open ('Merged_Diploid_A_GEN_Tetraploid_100.txt', 'w')

count = 0

with open ('Diploid_PIC_filtered.txt', 'rb') as csvfile1:

data1 = csv.reader(csvfile1,delimiter='\t')

heading1 = 0

for row1 in data1:

if heading1 == 0:

heading1 = 1

else:

f1 = row1[0]

f2 = int(row1[1])

flag = 0

with open ('Tetraploid_AGenome_RMindels.txt', 'rb') as csvfile2:

data2 = csv.reader(csvfile2,delimiter='\t')

heading2 = 0

for row2 in data2:

if heading2 == 0:

heading2 = 1

else:

if f2 - 100 <= int(row2[1]) and f2 + 100 >= int(row2[1]) and f1 == row2[0]:

count = count + 1

print count

#print f1, row2[0], f2, row2[1]

#fd.write(str(f1) + '\t')

#fd.write((f2) + '\t')

for i in xrange(0,5):

fd.write((row1[i]) + '\t')

for j in xrange(0,21):

fd.write((row2[j]) + '\t')

fd.write('\n')

fd.close()

**Script 4: Script to identify SNPs that are common to A genome & B tetraploids and A genome diploids(+/- 100bps)**

# This code is for filtering out SNPs that are common to A genome & B tetraploids and A genome diploids(+/- 100bps)

import csv

import numpy as np

fd = open ('Merged_Diploid_Tetraploid_100.txt', 'w')

count = 0

with open ('Diploid_PIC_filtered.txt', 'rb') as csvfile1:

data1 = csv.reader(csvfile1,delimiter='\t')

heading1 = 0

for row1 in data1:

if heading1 == 0:

heading1 = 1

else:

f1 = row1[0]

f2 = int(row1[1])

flag = 0

with open ('Merged_BLAST_final.txt', 'rb') as csvfile2:

data2 = csv.reader(csvfile2,delimiter='\t')

heading2 = 0

for row2 in data2:

if heading2 == 0:

heading2 = 1

else:

if f2 - 100 <= int(row2[1]) and f2 + 100 >= int(row2[1]) and f1 == row2[0]:

count = count + 1

print count

#print f1, row2[0], f2, row2[1]

#fd.write(str(f1) + '\t')

#fd.write((f2) + '\t')

for i in xrange(0,12):

fd.write((row1[i]) + '\t')

for j in xrange(0,50):

fd.write((row2[j]) + '\t')

fd.write('\n')

fd.close()

**Script 5: Script to correct for minor alleles; if MAF < 25%, call it a homozygote**

# This Code is to correct calls for minor alleles

import csv

import numpy as np

#pararameter to be varied

p = 0.25 #(25% in this case)

#first row title

first_row = True

fd = open ('Wholetarget_corrected.txt', 'w')

with open ('Wholetarget.txt', 'rb') as csvfile:

data = csv.reader(csvfile, delimiter='\t')

for row in data:

for idx in xrange(len(row)):

if first_row == True or idx < 8 or idx > 57:

fd.write(row[idx] + '\t')

else:

field = row[idx].split(':')

Ls = field[1].split(',')

if len(Ls) == 2:

l1 = int(Ls[0])

l2 = int(Ls[1])

band = float(l2) / (l1 + l2)

if 0<= band and band < p:

rslt = "0/0"

elif p <= band and band < 1-p:

rslt = "0/1"

else:

rslt = "1/1"

if rslt != field[0]:

field[0] = rslt

for field_elm in field:

fd.write(field_elm + ":")

fd.write('\t')

else:

fd.write(row[idx] + '\t')

first_row = False

fd.write('\n')

fd.close()

**Table S3.** Complete list of the targets and accessions used for targeted re-sequencing

| Target No | Target Name | Method | Target Genome |
| --- | --- | --- | --- |
| 1 | Aipa20:2730020 | SWEEP | B (Tetraploid) |
| 2 | Adur420_2:199475 | GATK | A (Diploid) & A/B (Tetraploid) |
| 3 | Adur105:427859 | GATK | A (Diploid) & A/B (Tetraploid) |
| 4 | comp75002_c0_seq1 | OLin | A (Diploid) & A/B (Tetraploid) |
| 5 | Adur97_1:1659452 | SWEEP | A (Tetraploid) |
| 6 | Aipa78_3:215435 | SWEEP | B (Tetraploid) |
| 7 | Adur39_1:1176326 | SWEEP | A (Tetraploid) |
| 8 | Aipa14:501305 | SWEEP | B (Tetraploid) |
| 9 | comp72966_c1_seq1 | OLin | A (Diploid) & A/B (Tetraploid) |
| 10 | comp72435 | OLin | B (Diploid) |
| 11 | comp69930 | OLin | B (Diploid) |
| 12 | Adur1176:44875 | GATK | A (Diploid) & A/B (Tetraploid) |
| 13 | comp76500_c2_seq1 | OLin | A (Diploid) & A/B (Tetraploid) |
| 14 | Aipa24_1:2950994 | SWEEP | B (Tetraploid) |
| 15 | comp74359_c0_seq1 | OLin | A (Diploid) & A/B (Tetraploid) |
| 16 | Adur63:248218 | GATK | A (Diploid) & A/B (Tetraploid) |
| 17 | comp73108 | OLin | B (Diploid) |
| 18 | Adur35_1:176390 | GATK | A (Diploid) & A/B (Tetraploid) |
| 19 | Adur184:363976 | GATK | A (Diploid) & A/B (Tetraploid) |
| 20 | comp72236 | OLin | B (Diploid) |
| 21 | comp75754_c0_seq15 | OLin | A (Diploid) & A/B (Tetraploid) |
| 22 | comp76672_c1_seq2 | OLin | A (Diploid) & A/B (Tetraploid) |
| 23 | Adur43:2486439 | GATK | A (Diploid) & A/B (Tetraploid) |
| 24 | comp75852_c1_seq2 | OLin | A (Diploid) & A/B (Tetraploid) |
| 25 | Adur6_1:1261808 | GATK | A (Diploid) & A/B (Tetraploid) |
| 26 | Adur2_3_3190021 | SWEEP | A (Tetraploid) |
| 27 | AdurA04:120754095 | SNP Chip | A/B (Tetraploid) |

(Table S3 continued)

| Target No | Target Name | Method | Target Genome |
| --- | --- | --- | --- |
| 28 | Adur169:519284 | GATK | A (Diploid) & A/B (Tetraploid) |
| 29 | comp76156_c0_seq1 | OLin | A (Diploid) & A/B (Tetraploid) |
| 30 | Adur313:17300 | GATK | A (Diploid) & A/B (Tetraploid) |
| 31 | Adur18_4:644142 | GATK | A (Diploid) & A/B (Tetraploid) |
| 32 | Aradu.A02:40760413 | SNP Chip | A (Tetraploid) |
| 33 | Adur2_2:2128866 | GATK | A (Diploid) & A/B (Tetraploid) |
| 34 | Aradu.A04:120888870 | SNP Chip | A/B (Tetraploid) |
| 35 | Adur126:1822782 | GATK | A (Diploid) & A/B (Tetraploid) |
| 36 | comp71231_c0_seq1 | OLin | A (Diploid) & A/B (Tetraploid) |
| 37 | Araip.B05:25231571 | SNP Chip | B (Tetraploid) |
| 38 | Adur199:428032 | GATK | A (Diploid) & A/B (Tetraploid) |
| 39 | Aradu.A03:27606767 | SNP Chip | A (Tetraploid) |
| 40 | Adur122:1491484 | GATK | A (Diploid) & A/B (Tetraploid) |
| 41 | Aipa58:6370858 | SWEEP | B (Tetraploid) |
| 42 | Adur675:242530 | GATK | A (Diploid) & A/B (Tetraploid) |
| 43 | Adur401:117599 | GATK | A (Diploid) & A/B (Tetraploid) |
| 44 | comp75435_c2_seq3:1785 | OLin | A (Diploid) & A/B (Tetraploid) |
| 45 | Adur18_4:644142 | GATK | A (Diploid) & A/B (Tetraploid) |
| 46 | AdurA03:27434672 | SNP Chip | A (Diploid) & A/B (Tetraploid) |
| 47 | Adur79:823475 | SWEEP | A (Tetraploid) |
| 48 | Adur115:324995 | GATK | A (Diploid) & A/B (Tetraploid) |

**Table S4.** Complete list of primers used for Access Array experiment

| Primer No. | Primer Name | Forward Specific Sequence | Reverse Specific Sequence |
| --- | --- | --- | --- |
| 1 | Aipa20:2730020 | TAGCAGCAGCTTCACCTTCA | GACTCACGCTTCTGTGTGGA |
| 2 | Adur420_2:199475 | CACGGGGAAGGAATATGCTA | TAAAGAAAACTGCCGGATGG |
| 3 | Adur105:427859 | CGGTGCTGTATGGGGTACAT | GCTCTCATGGCTGGTTTAGC |
| 4 | comp75002_c0_seq1:2837 | GTGGATCTTCATCCCAAAGC | GGTTGGTGGAAGGTCAAGAA |
| 5 | Adur97_1:1659452 | CACCTTTCAGCTTTGCTTCC | GAAATGGGTGAGGAGGTGAA |
| 6 | Aipa78_3:215435 | TGATGCTGCACCTTTCAAAC | GCGACCACTTCCAAATTGTT |
| 7 | Adur39_1:1176326 | CAAGAAAGAGCCCTGTGAGG | AGACTGCACTCCCCATCAAC |
| 8 | Aipa14:501305 | CTAACACGGTGGTTGGACCT | ACCCTAGCCGCCATTAGTTT |
| 9 | comp72966_c1_seq1:1115 | TAGCAGCGGAAGGACAAGAT | GCATCAGGTGACCAACCTTT |
| 10 | comp72435_c0_seq1 | ACACCGATTCCGACAAGTTC | CGTTGCTTCAACTCCCTCTC |
| 11 | comp69930_c2_seq1 | TCCAAATTGCCCACAAAAAT | GAGCGATTATGCCAAATGGT |
| 12 | Adur1176_44875 | ATCCTCCTTTGAATGGCAAG | CCTCATGGTTGAAGCTAGGG |
| 13 | comp76500_c2_seq1:2003 | GAGCACTCAATGCGTGAAAA | CAGCACGGGAAGAAGATTGT |
| 14 | Aipa24_1:2950994 | GCTCTATCCTTTGCGTCGTC | TCAAAATCCCCGTTACTTGG |
| 15 | comp74359_c0_seq1:3194 | ACTTGGGGCTTTCCAACTTT | GAAGCCAAGGTGGTGATGAT |
| 16 | Adur63:248218 | AAAAGCAGCACCCTTTCTCA | TGAATGGAGGTTCACGATCA |
| 17 | comp73108_c0_seq1:1799 | CAGAAGTTTGCACGGACTCA | GTAGAGCGGCTGCTTCAATC |
| 18 | Adur35_1:176390 | CCCTCGACAATGGTGAGTTT | TCATGGCCGTCTTCACTTTC |
| 19 | Adur184:363976 | GGCGTCATAGTGGGAGTCAT | GCCAGCATCAACAGCTTACA |
| 20 | comp72236_c2_seq1:1273 | AGCGCAAGAAAAAGGTGAAG | ATTCCATTGGGGACTTTTCC |
| 21 | comp75754_c0_seq15:997 | GTGGTGGCTTCAGAGAAAGG | GTATTGCCTTGATGGCCTTG |
| 22 | comp76672_c1_seq2:803 | ATGTCTGGCCTCACAAGGAA | CACTGTTGGCCAGTCTTCAA |
| 23 | Adur43:2586439 | TCAGCTGGAATTGGAGCAACT | CCCCATGACTCCAGTGTGAC |
| 24 | comp75852_c1_seq2:5024 | CATGCCAGCAAGTTTCTTCA | GGTCCACCCTGCTTGATTTA |
| 25 | Adur6_1:1261808 | AGAGCCACCTTAGCCTCCTC | AAACGATAAAGTGGCCAACG |
| 26 | Adur2_3:3190021 | CGACGAGAAGGTTCTGAAGG | ACACTTGGCTTCCCCTTTTT |
| 27 | AdurA04:120754095_  AipaB04:13094579 | GGAGTGCAGCATCAAGACTAA | CCTGCAGAGGGTTTGTTCTC |

**(**Table S4 continued)

| Primer No. | Primer Name | Forward Specific Sequence | Reverse Specific Sequence |
| --- | --- | --- | --- |
| 28 | Adur169:519284 | CGAAAGCATGGGTCAAAACT | AATTGAAGGACGTGGCAAAG |
| 29 | comp76156_c0_seq1:8035 | CATGCTTTGGCCTTCAGAAT | CTCTTCAGCCGGTGTCATCT |
| 30 | Adur313:17300 | AATTCGGAACCTCTTTAGGG | ACTGCGTGGCTCTGAAATCT |
| 31 | Adur18_4:644142 | ACCCGAAATTGTACATGGCA | TGCGCCAAGATGAGAGAGAA |
| 32 | Aradu.A02:40760413 | CGTGCAACAATTCCAATGAG | GGCATCAATCAACGATGCTA |
| 33 | Adur2_2:2128866 | TGCTATGCACCCTTTTAGGC | GTATCCGACGAGGAGATGGA |
| 34 | AdurA04:120888870_  AipaB04:131009220 | CGGTGGAATCACTTTGGAAT | CCAGGACAAAACCAATCACC |
| 35 | Adur126:1822782 | TCTGACTTAATTATGGCTTGGTG | TGGCACCAAAAACTTCAATG |
| 36 | comp71231_c0_seq1:2588 | TGTCATGGAATCCACTGTGC | TCCTGCTCAGGATCCAATTC |
| 37 | Araip.B05:25231571 | CCGGCAGATCTCTACCAGTC | AGCATGCTACTGTGATTCTTGG |
| 38 | Adur199:428032 | TTCAATTGGCAACTCCCCGA | TCCCCTCTCTGGTGTAAAGGA |
| 39 | Aradu.A03:27606767 | GAGGGTGCCTAGCCTTGTATT | GCATCTTGCTCACCCCTTTA |
| 40 | Adur122:1491484 | CCTTGCTTGTTGGGAGATGT | TCGCTTTTCAGCTCCGTATT |
| 41 | Aipa58:6370858 | CCAGATTTTTCCATGGTGCT | AGAGACTGTGGTCCCTGGTG |
| 42 | Adur675:242529 | ACCTCTTTGCACACCTTGCT | GGCTTGCACAATCTCACTCA |
| 43 | Adur401:117599 | GGAATTGGACCCATATGACG | ATTCCTCATCCGCTACAAGC |
| 44 | comp75435_c2_seq3:1936 | TGCATGCAAGTGTTTGGTTT | ACACTTGCCAGGGAAATCAG |
| 45 | Adur18_4:644192 | TTTGACCATTTGGTTTTCTCG | CCAGGGCTTCCAAGATAATG |
| 46 | AdurA03:27434672 | TCATGCACACAATGGAGACA | GCGGGCAGGTAAGTTATTGA |
| 47 | Adur79:823475 | GCAGGAGTTTCCACCAGGTA | AGGCCTTTGTCTTGCTTGAA |
| 48 | Adur115:324993 | TTCCCATAGATGAGGTGAGGA | ATCTTGCTGACCACCTTGCT |

**Table S5.** Complete list of accessions used for targeted re-sequencing

| Sr. No. | Accessions | Botanical type | References |
| --- | --- | --- | --- |
| 1 | CC155A | Peruviana | [41] |
| 2 | CC227 | Peruviana | [41] |
| 3 | CC367 | Virginia | [41] |
| 4 | CC559 | Valencia | [41] |
| 5 | CC230 | Runner | [41] |
| 6 | CC270 | Runner | [41] |
| 7 | CC157 | Aequatoriana | [41] |
| 8 | PI 648241 | Hirsuta | - |
| 9 | BSS56 | Spanish | [40] |
| 10 | Jupiter | Virginia | - |
| 11 | New Mexico Valencia C | Valencia | [42] |
| 12 | OLin | Spanish | [43] |
| 13 | TamrunOL07 | Runner | [44] |
| 14 | UF439-16-10-3-2 | Runner | [45] |
| 15 | Nama | Runner | - |
| 16 | GM656 | Spanish | - |
| 17 | PC79-79 | Spanish | - |
| 18 | NkatieSARI | Virginia | [46] |
| 19 | Schubert | Spanish | [47] |
| 20 | TxAG-6 | Synthetic | [48] |
| 21 | Tx071304 | Runner | - |
| 22 | TxL080243-06 | Runner | - |
| 23 | OLé | Spanish | [49] |
| 24 | TamValOL14 | Valencia | [50] |
| 25 | Venus | Virginia | [51] |
| 26 | 55-437 | Spanish | - |
| 27 | 60-02-03-02 | Interspecific BC3 | - |
| 28 | 43-09-03-02 | Interspecific BC3 | - |
| 29 | TxL090105-07 | Virginia | - |

**(**Table S5 Continued)

| Sr. No. | Accessions | Botanical type | References |
| --- | --- | --- | --- |
| 30 | NC-7 | Virginia | [52] |
| 31 | TxL080256-02 | Runner | - |
| 32 | WWT1362 | Aequatoriana | - |
| 33 | NuMex-1 | Valencia | [53] |
| 34 | New Mexico Valencia A | Valencia | [42] |
| 35 | 308-TAN | - | - |
| 36 | GKP10017 | *A. cardenasii* | [54] |
| 37 | KSSc36024 | *A. cruziana* | [54] |
| 38 | GK10602 | *A. diogoi* | [54] |
| 39 | K7988 | *A. duranensis* | [54] |
| 40 | KSSc38901 | *A. duranensis* | [54] |
| 41 | GKPSSc30076 | *A. ipaensis* | [54] |
| 42 | GKSSc30097 | *A. magna* | [54] |
| 43 | K9484 | *A. batizicoi* | [54] |
| 44 | 12943 | *A. dardanii* | - |
| 45 | 12946 | *A. dardanii* | - |
| 46 | 9902-1 | *A. valsii* | - |
| 47 | 9646 | *A. paraguarensis* | - |
| 48 | VSGR 6416 | *A. praecox* | - |

**Table S6.**  Fluidigm protocols used.

Preamplification protocol

| Temperature | Time | Cycles | Description |
| --- | --- | --- | --- |
| 95 ^o^c | 15 min |  | Hold |
| 95 ^o^c | 15 sec | 14 | Denaturation |
| 60 ^o^c | 8 min |  | Annealing/extension |
| 4 ^o^c | ∞ |  | Hold |

Preparation of Assay Pre-Mix

| Component | Volume per assay solutions (μl) | Volume for 48 assay pools with overage (μl) |
| --- | --- | --- |
| TSP (Targeted Sequence library Preparation) Assay loading reagent | 2.5 | 150 |
| PCR water | 27.5 | 1650 |
| Total | 30.0 | 1800 |

Preparation of Sample Pre-Mix. (All reagents were used at the concentration provided in the kit).

| Component | Volume per Assay Solution (μL) |
| --- | --- |
| PCR quality Water | 33.6 |
| 4X (TSP) Master Mix | 90.0 |
| TSP Sample Loading Reagent | 18.0 |
| TSP DNA Polymerase | 14.4 |
| Total | 156.0 |

Preparation of 20X Assay Pool

| Component | Volume per Assay Solution (μL) |
| --- | --- |
| Assay Pre-Mix (From Table **S3.C.**) | 30 |
| Pooled forward and reverse primers (PCR water for unused inlets) | 20 |
| Total | 50 |

Preparation of Sample Mix

| Component | Volume per Assay Solution (μL) |
| --- | --- |
| Sample pre-mix | 2 |
| Genomic DNA sample | 2 |
| Barcode primer from TSP Barcode plate | 1 |
| Total | 5 |

Thermal cycling conditions for the Fluidigm FC1 cycler

| Description | Cycles | Temperature | Time |
| --- | --- | --- | --- |
| Thermal mixing | 1 | 65 | 1,083 sec |
| Hot start | 1 | 95 | 870 sec |
|  | 1 | 97 | 30 sec |
| PCR | 17 | 97 | 15 sec |
|  |  | 60 | 90 sec |
|  |  | 68 | 90 sec |
| Final extension | 1 | 68 | 180 sec |
| Hold | 1 | 4 | Until harvest |

**Table S7.** KASP experiment details


KASP - Preparation of primer assay mix.

| **Ingredient** | **Volume (μl)** |
| --- | --- |
| Primer X 100 μM stock* | 10 |
| Primer Y 100 μM stock* | 10 |
| Primer C 100 μM stock* | 30 |
| PCR water | 50 |
| **Total** | **100** |

*Prepare primer stock by diluting to 100 μM in TE buffer.

KASP- Reaction recipe. Full reaction for 96-well plate, half reaction for 384-well plate.

| **Ingredient** | **Volume for full reaction (μl)** | **Volume for half reaction (μl)** |
| --- | --- | --- |
| KASP master mix | 4.00 | 2.00 |
| Primer assay mix | 0.15 | 0.08 |
| PCR water | 1.85 | 0.92 |
| **Subtotal** | **6.00** | **3.00** |
| DNA | 2.00 | 1.00 |
| **Total** | **8.00** | **4.00** |

Light Cycler PCR program.

| **Program** | **Cycles (n)** | **Step** | **Temp (°C)** | **Time (mm:ss)** |
| --- | --- | --- | --- | --- |
| Denaturation | 1 | 1 | 95 | 15:00 |
| Touchdown | 10 | 1 | 95 | 0:20 |
|  |  | 2 | 65 (-0.8/cycle) | 1:00 |
| Amplification | 25 | 1 | 95 | 0:20 |
|  |  | 2 | 57 | 1:00 |
| Cooling | 1 | 1 | 37 | 1:00 |
| Quantification | 2 | 1 | 37 | 0:05 |


**Table S.8.** KASP primer sequences

| Primer No. | Primer Sequence | | |
| --- | --- | --- | --- |
| Primer Type | Allele flanking C | Allele-specific (X) | Allele-specific (Y) |
| P01 | TGATGCATATAAGCTAATCAAGGTC | CAGGAACCCGAGCGTG | ACAGGAACCCGAGCGTC |
| P03 | TCGCATTATCGGGAAAATAAA | GTGAGGTGTTGGAGAAAGCTG | GTTAAATATTCTTCATCGTAGTAAGGGC |
| P04 | CCAGATTTTTCCATGGTGCT | CAAACAAGGAGGGAGCAGTTA | AAACAAGGAGGGAGCAGTTG |
| P05 | TACCTCCCCTTCTTTCTTGC | CGCAAGAAGAAGAGTGCAAA | TCGCAAGAAGAAGAGTGCAAC |
| P06 | TGATGCATATAAGCTAATCAAGGTC | CAGGAACCCGAGCGTG | ACAGGAACCCGAGCGTC |
| P07 | ACAGGAACCCGAGCGTC | GGTGACCAAGAACCCCACT | GGTGACCAAGAACCCCACT |
| P08 | TGGTCTTAACTAAAGGGCATCAA | CAACCTCAATTCACTTCCAAAAATA | AACCTCAATTCACTTCCAAAAATC |
| P10 | CATCGTCAAGAACCAGAAGGA | GATATAAAACAAAGTCTCCGCAATAAG | GATATAAAACAAAGTCTCCGCAATAAG |

(For Primer name refer to **Table S4.**)

**Table S9.** Summary of percentage heterozygotes in SNP calls. Data are from 46 accessions before and after dropping putative heterozygotes with less than 20% minor allele frequency.

(To identify the heterozygous SNPs, the number of 0/1 calls were calculated for each accession. If the total number of reads for minor allele was more than 20% of the total number of reads for major allele or if the total number of reads for major allele was less than 20% of the total number of reads for minor allele, the 0/1 call was changed to 0/0 or 1/1).

| Accessions | % Heterozygotes before correction | % Heterozygotes after correction |
| --- | --- | --- |
| *A. duranensis* 7988 | 9.0 | 0.2 |
| *A. batizicoi* 9484 | 21.2 | 0.3 |
| *A. paraguaensis* 9646 | 13.9 | 0.3 |
| *A. cardenasii* 10017 | 9.9 | 0.3 |
| *A. diogoi* 10602 | 11.9 | 0.2 |
| 12493 | 13.6 | 0.2 |
| 12496 | 14.4 | 0.3 |
| *A. ipaensis* 30076 | 3.3 | 0.1 |
| *A. magna* 30097 | 9.0 | 0.3 |
| *A. cruziana* 36024 | 10.9 | 0.2 |
| *A. duranensis* 38901 | 7.3 | 0.3 |
| 308-TAN | 8.8 | 0.2 |
| 43-09-03-02 | 7.1 | 0.1 |
| 55-437 | 10.2 | 0.2 |
| 60-02-03-02 | 9.7 | 0.2 |
| 9902-1 | 9.5 | 0.3 |
| *A. praecox* | 8.9 | 0.2 |
| BSS56 | 6.4 | 0.1 |
| CC155A | 4.6 | 0.1 |
| CC157 | 8.7 | 0.1 |
| CC227 | 6.4 | 0.2 |
| CC230 | 6.9 | 0.2 |
| CC270 | 7.9 | 0.2 |
| CC367 | 8.1 | 0.2 |
| CC559 | 4.9 | 0.1 |
| GM656 | 9.6 | 0.2 |
| Jupiter | 6.7 | 0.2 |
| NkatieSARI | 6.6 | 0.2 |
| Nama | 7.0 | 0.2 |
| NC7 | 6.4 | 0.1 |
| NmValA | 6.0 | 0.1 |
| NmValC | 6.7 | 0.1 |
| NuMex1 | 5.7 | 0.1 |
| Olin | 7.4 | 0.1 |
| PC79-79 | 11.5 | 0.3 |
| PI48241 | 4.2 | 0.1 |
| Schubert | 9.0 | 0.2 |
| TamrunOL07 | 5.3 | 0.1 |
| Tx090105-07 | 5.9 | 0.2 |
| TxAG6 | 24.4 | 0.4 |
| TxL080243-06 | 7.1 | 0.1 |
| TxL080287-05 | 4.9 | 0.1 |
| UF439 | 5.7 | 0.2 |
| Venus | 6.8 | 0.2 |
| WWT1632 | 5.2 | 0.1 |
| Mean % Heterozygotes | 8.46 | 0.18 |

**Table S10.** Estimated error percentages for MiSeq for each base pair substitutions from barcode regions of accessions OLin and Jupiter

| **Substitution** | **Error (%)** |
| --- | --- |
| T/A | 0.33 |
| T/G | 0.69 |
| T/C | 0.17 |
| A/T | 0.84 |
| A/G | 0.11 |
| A/C | 0.19 |
| G/T | 0.40 |
| G/A | 1.38 |
| G/C | 0.14 |
| C/T | 0.12 |
| C/A | 0.14 |
| C/G | 0.03 |

**Table S11. Blast results for all targets showing mapping against possible paralogous sequence matches** (query sequence: A diploid genome, for which the e-value = 10.0)

| **Target No.** | **No. of paralogous matches** | **Paralogous match** | **E-value** |
| --- | --- | --- | --- |
| 1 | 3 | aradu.Aradu.A02 | 0.74 |
|  |  | aradu.Aradu.A03 | 0.74 |
|  |  | aradu.Aradu.A05 | 0.74 |
| 2 | 2 | aradu.Aradu.A02 | 0.74 |
|  |  | aradu.Aradu.A05 | 0.004 |
| 3 | 0 |  |  |
| 4 | 2 | aradu.Aradu.A07 | 0.74 |
|  |  | aradu.Aradu.A10 | 0.74 |
| 5 | 4 | aradu.Aradu.A02 | 0.004 |
|  |  | aradu.Aradu.A03 | 0.74 |
|  |  | aradu.Aradu.A06 | 0.004 |
|  |  | aradu.Aradu.A09 | 0.74 |
| 6 | 1 | aradu.Aradu.A03 | 0.74 |
| 7 | 2 | aradu.Aradu.A04 | 0.19 |
|  |  | aradu.Aradu.A10 | 0.19 |
| 8 | 16 | aradu.Aradu.A02 | 0.74 |
|  |  | aradu.Aradu.A02 | 0.74 |
|  |  | aradu.Aradu.A02 | 0.74 |
|  |  | aradu.Aradu.A02 | 0.74 |
|  |  | aradu.Aradu.A03 | 0.74 |
|  |  | aradu.Aradu.A04 | 0.74 |
|  |  | aradu.Aradu.A04 | 0.74 |
|  |  | aradu.Aradu.A05 | 0.74 |
|  |  | aradu.Aradu.A05 | 0.74 |
|  |  | aradu.Aradu.A06 | 0.74 |
|  |  | aradu.Aradu.A08 | 0.74 |
|  |  | aradu.Aradu.A08 | 0.74 |
|  |  | aradu.Aradu.A08 | 0.74 |
|  |  | aradu.Aradu.A09 | 0.74 |
|  |  | aradu.Aradu.A10 | 0.74 |
|  |  | aradu.Aradu.A02 | 0.74 |
| 9 | 0 |  |  |
| 10 | 0 |  |  |
| 11 | 3 | aradu.Aradu.A02 | 0.19 |
|  |  | aradu.Aradu.A03 | 0.74 |
|  |  | aradu.Aradu.A04 | 0.004 |
| 12 | 0 |  |  |
| 13 | 0 |  |  |
| 14 | 0 |  |  |

(Table S11 continued)

| Target No. | No. of paralogous matches | Paralogous match | E-value |
| --- | --- | --- | --- |
| 15 | 1 | aradu.Aradu.A06 | 0.19 |
| 16 | 4 | aradu.Aradu.A01 | 0.74 |
|  |  | aradu.Aradu.A02 | 0.74 |
|  |  | aradu.Aradu.A04 | 0.74 |
|  |  | aradu.Aradu.A06 | 0.74 |
| 17 | 0 |  |  |
| 18 | 8 | aradu.Aradu.A01 | 0.004 |
|  |  | aradu.Aradu.A01 | 0.004 |
|  |  | aradu.Aradu.A01 | 0.004 |
|  |  | aradu.Aradu.A01 | 0.004 |
|  |  | aradu.Aradu.A01 | 0.19 |
|  |  | aradu.Aradu.A01 | 0.19 |
|  |  | aradu.Aradu.A01 | 0.19 |
|  |  | aradu.Aradu.A07 | 0.19 |
| 19 | 1 | aradu.Aradu.A06 | 0.004 |
| 20 | 7 | aradu.Aradu.A06 | 0.19 |
|  |  | aradu.Aradu.A06 | 0.19 |
|  |  | aradu.Aradu.A07 | 0.19 |
|  |  | aradu.Aradu.A07 | 0.19 |
|  |  | aradu.Aradu.A07 | 0.19 |
|  |  | aradu.Aradu.A08 | 0.19 |
|  |  | aradu.Aradu.A08 | 0.19 |
| 21 | 1 | aradu.Aradu.A06 | 0.051 |
| 22 | 3 | aradu.Aradu.A02 | 0.004 |
|  |  | aradu.Aradu.A02 | 0.004 |
|  |  | aradu.Aradu.A02 | 0.19 |
| 23 | 4 | aradu.Aradu.A03 | 3.4 |
|  |  | aradu.Aradu.A04 | 3.4 |
|  |  | aradu.Aradu.A08 | 3.4 |
|  |  | aradu.Aradu.A09 | 3.4 |
| 24 | 13 | aradu.Aradu.A01 | 0.74 |
|  |  | aradu.Aradu.A01 | 0.74 |
|  |  | aradu.Aradu.A01 | 0.74 |
|  |  | aradu.Aradu.A01 | 0.74 |
|  |  | aradu.Aradu.A04 | 0.74 |
|  |  | aradu.Aradu.A04 | 0.74 |
|  |  | aradu.Aradu.A04 | 0.74 |
|  |  | aradu.Aradu.A06 | 0.74 |
|  |  | aradu.Aradu.A06 | 0.74 |
|  |  | aradu.Aradu.A06 | 0.74 |
|  |  | aradu.Aradu.A07 | 0.74 |

(Table S11 continued)

| Target No. | No. of paralogous matches | Putative paralogous match | E-value |
| --- | --- | --- | --- |
|  |  | aradu.Aradu.A08 | 0.74 |
|  |  | aradu.Aradu.A10 | 0.74 |
| 25 | 0 |  |  |
| 26 | 4 | aradu.Aradu.A01 | 0.9 |
|  |  | aradu.Aradu.A06 | 3.4 |
|  |  | aradu.Aradu.A07 | 0.24 |
|  |  | aradu.Aradu.A10 | 0.90 |
| 27 | 20 | aradu.Aradu.A03 | 0.24 |
|  |  | aradu.Aradu.A03 | 0.9 |
|  |  | aradu.Aradu.A04 | 0.004 |
|  |  | aradu.Aradu.A04 | 0.004 |
|  |  | aradu.Aradu.A04 | 0.062 |
|  |  | aradu.Aradu.A04 | 0.24 |
|  |  | aradu.Aradu.A04 | 0.24 |
|  |  | aradu.Aradu.A04 | 0.24 |
|  |  | aradu.Aradu.A04 | 0.9 |
|  |  | aradu.Aradu.A04 | 0.9 |
|  |  | aradu.Aradu.A04 | 0.9 |
|  |  | aradu.Aradu.A04 | 0.9 |
|  |  | aradu.Aradu.A04 | 0.9 |
|  |  | aradu.Aradu.A04 | 0.9 |
|  |  | aradu.Aradu.A01 | 3.4 |
|  |  | aradu.Aradu.A01 | 3.4 |
|  |  | aradu.Aradu.A05 | 0.24 |
|  |  | aradu.Aradu.A05 | 0.9 |
|  |  | aradu.Aradu.A06 | 3.4 |
|  |  | aradu.Aradu.A09 | 3.4 |
| 28 | 2 | aradu.Aradu.A03 | 3.4 |
|  |  | aradu.Aradu.A05 | 0.016 |
| 29 | 0 |  |  |
| 30 | 1 | aradu.Aradu.A04 | 0.9 |
| 31 | 2 | aradu.Aradu.A03 | 0.016 |
|  |  | aradu.Aradu.A07 | 3.4 |
| 32 | 10 | aradu.Aradu.A03 | 3.4 |
|  |  | aradu.Aradu.A04 | 3.4 |
|  |  | aradu.Aradu.A04 | 3.4 |
|  |  | aradu.Aradu.A04 | 3.4 |
|  |  | aradu.Aradu.A04 | 3.4 |
|  |  | aradu.Aradu.A04 | 3.4 |

(Table S11 continued)

| Target No. | No. of paralogous matches | Paralogous match | E-value |
| --- | --- | --- | --- |
|  |  | aradu.Aradu.A06 | 3.4 |
|  |  | aradu.Aradu.A07 | 3.4 |
|  |  | aradu.Aradu.A08 | 3.4 |
|  |  | aradu.Aradu.A10 | 3.4 |
| 33 | 1 | aradu.Aradu.A05 | 3.4 |
| 34 | 6 | aradu.Aradu.A02 | 0.24 |
|  |  | aradu.Aradu.A02 | 0.9 |
|  |  | aradu.Aradu.A04 | 0.016 |
|  |  | aradu.Aradu.A06 | 3.4 |
|  |  | aradu.Aradu.A07 | 3.4 |
|  |  | aradu.Aradu.A09 | 3.4 |
| 35 | 3 | aradu.Aradu.A06 | 5.1 |
|  |  | aradu.Aradu.A09 | 1.3 |
|  |  | aradu.Aradu.A10 | 1.3 |
| 36 | 0 |  |  |
| 37 | 0 |  |  |
| 38 | 4 | aradu.Aradu.A01 | 3.4 |
|  |  | aradu.Aradu.A05 | 0.016 |
|  |  | aradu.Aradu.A09 | 3.4 |
|  |  | aradu.Aradu.A10 | 0.24 |
| 39 | 35 | aradu.Aradu.A02 | 0.24 |
|  |  | aradu.Aradu.A02 | 0.24 |
|  |  | aradu.Aradu.A03 | 0.24 |
|  |  | aradu.Aradu.A04 | 0.04 |
|  |  | aradu.Aradu.A04 | 0.04 |
|  |  | aradu.Aradu.A04 | 0.04 |
|  |  | aradu.Aradu.A04 | 0.04 |
|  |  | aradu.Aradu.A04 | 0.04 |
|  |  | aradu.Aradu.A04 | 0.016 |
|  |  | aradu.Aradu.A04 | 0.062 |
|  |  | aradu.Aradu.A04 | 0.24 |
|  |  | aradu.Aradu.A04 | 0.24 |
|  |  | aradu.Aradu.A04 | 0.24 |
|  |  | aradu.Aradu.A04 | 0.24 |
|  |  | aradu.Aradu.A04 | 0.24 |
|  |  | aradu.Aradu.A04 | 0.24 |
|  |  | aradu.Aradu.A04 | 0.24 |
|  |  | aradu.Aradu.A04 | 0.24 |
|  |  | aradu.Aradu.A04 | 0.24 |
|  |  | aradu.Aradu.A04 | 0.24 |
|  |  | aradu.Aradu.A04 | 0.24 |

(Table S11 continued)

| Target No. | No. of paralogous matches | Paralogous match | E-value |
| --- | --- | --- | --- |
|  |  | aradu.Aradu.A04 | 0.24 |
|  |  | aradu.Aradu.A04 | 0.24 |
|  |  | aradu.Aradu.A04 | 0.24 |
|  |  | aradu.Aradu.A04 | 0.24 |
|  |  | aradu.Aradu.A04 | 0.24 |
|  |  | aradu.Aradu.A04 | 0.24 |
|  |  | aradu.Aradu.A04 | 0.24 |
|  |  | aradu.Aradu.A04 | 0.24 |
|  |  | aradu.Aradu.A04 | 0.24 |
|  |  | aradu.Aradu.A04 | 0.24 |
|  |  | aradu.Aradu.A04 | 0.9 |
|  |  | aradu.Aradu.A04 | 3.4 |
|  |  | aradu.Aradu.A04 | 3.4 |
|  |  | aradu.Aradu.A08 | 0.004 |
| 40 | 4 | aradu.Aradu.A05 | 3.4 |
|  |  | aradu.Aradu.A06 | 3.4 |
|  |  | aradu.Aradu.A07 | 3.4 |
|  |  | aradu.Aradu.A09 | 3.4 |
| 41 | 1 | aradu.Aradu.A10 | 3.4 |
| 42 | 1 | aradu.Aradu.A02 | 0.016 |
| 43 | 0 |  |  |
| 44 | 3 | aradu.Aradu.A02 | 3.4 |
|  |  | aradu.Aradu.A05 | 3.4 |
|  |  | aradu.Aradu.A10 | 3.4 |
| 45 | 9 | aradu.Aradu.A04 | 3.4 |
|  |  | aradu.Aradu.A04 | 3.4 |
|  |  | aradu.Aradu.A05 | 3.4 |
|  |  | aradu.Aradu.A06 | 3.4 |
|  |  | aradu.Aradu.A07 | 3.4 |
|  |  | aradu.Aradu.A08 | 3.4 |
|  |  | aradu.Aradu.A09 | 3.4 |
|  |  | aradu.Aradu.A10 | 3.4 |
|  |  | aradu.Aradu.A10 | 3.4 |
| 46 | 1 | aradu.Aradu.A08 | 3.4 |
| 47 | 0 |  |  |
| 48 | 1 | aradu.Aradu.A03 | 3.4 |
| **Total** | **183** |  |  |

**Table S12.** Example of Clustal alignment. The figure shows the alignment of the paralog match Aradu.A01 and Araip.B04 for the target Adur420_2:199475 (The circled variant represents the target SNP).


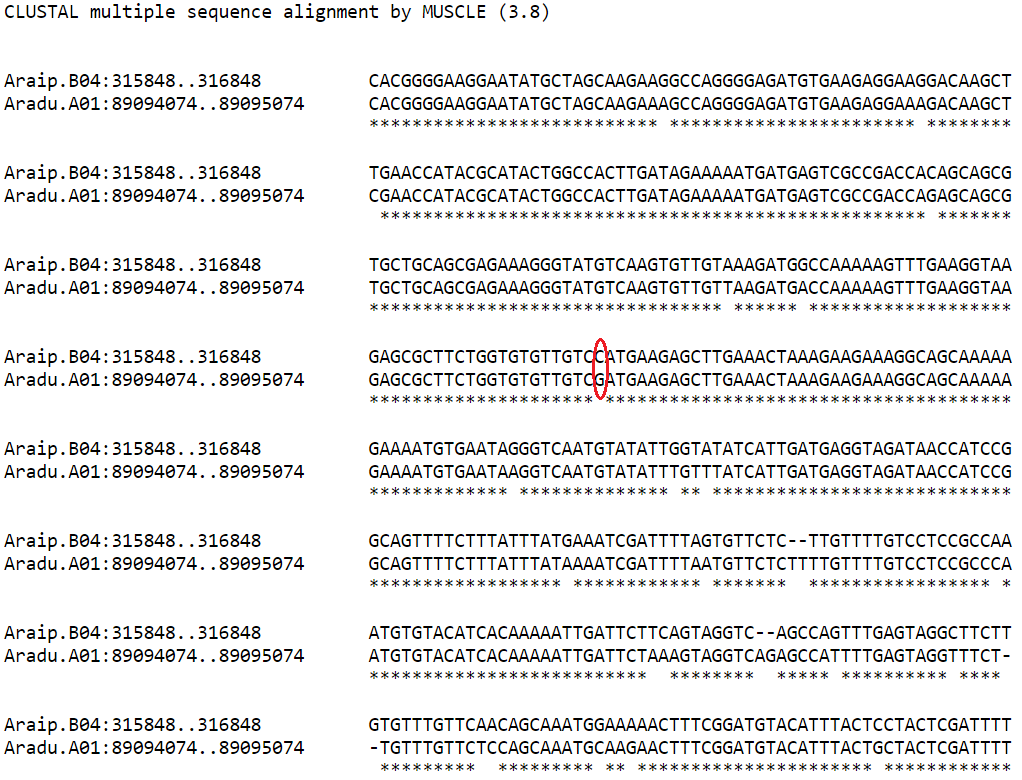

Supplement: Supplementary file 1 [file genes-11-01220-s001.zip › Supplementary tables_Oct2020_rev.docx]
